# Predictive value of cardiac magnetic resonance for the diagnosis and surgical relief of pericardial constriction

**DOI:** 10.1186/1532-429X-15-S1-P107

**Published:** 2013-01-30

**Authors:** Kate Hanneman, Hadas Moshonov, Rachel M Wald, Elsie T Nguyen, Kim A Connelly, Andrew M Crean

**Affiliations:** 1 Department of Medical Imaging, University Health Network, University of Toronto, Toronto, ON, Canada; 2 Division of Cardiology, Peter Munk Cardiac Centre, Toronto General Hospital, University Health Network, University of Toronto, Toronto, ON, Canada; 3 Division of Cardiology, St. Michael's Hospital, University of Toronto, Toronto, ON, Canada

## Background

The diagnosis of pericardial constriction (PC) remains challenging and cardiac magnetic resonance (CMR) is increasingly used as a diagnostic tool. The objective of this study was to evaluate CMR findings for the prediction of subsequent surgical pericardiectomy.

## Methods

CMR studies of 36 patients referred to assess for PC were evaluated retrospectively. Patients were divided into two groups depending on whether they subsequently had their pericardium stripped (n=18) or did not (n=18). IVC and aortic areas were determined by manual contouring on a single axial-SSFP image in maximum systole at the level of the esophageal hiatus. The ratio of IVC to aortic (I:A) area was calculated. Cross-sectional areas were indexed to body surface area (BSA). Quantitative data was assessed with a two-sample t-test and qualitative data was assessed with Fisher's exact test. A logistic regression model was used to determine the predictive probability of surgical pericardiectomy based on CMR features. Odds ratios (ORs) were calculated and receiver operating characteristic (ROC) analysis was performed.

## Results

**Figure 1:**
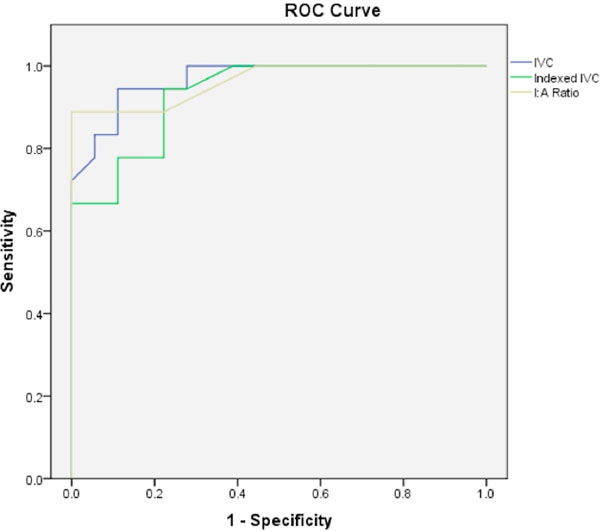
ROC curves for IVC cross-sectional area (blue), indexed IVC cross-sectional area (green) and IVC to aortic ratio (yellow). Area under the curve (AUC) and 95% confidence intervals are 0.968 (0.92, 1.00), 0.932 (0.86, 1.00) and 0.963 (0.91, 1.00) respectively.

Mean age of patients was 53.9±15.3 years, 72% (n=26) male, with no significant difference in mean age between the two groups (p=0.429). In patients with constriction, the underlying etiology was idiopathic (39%, n=7), infectious (28%, n=5), post-surgical (17%, n=3), connective-tissue disease (11%, n=2), and post-radiation (6%, n=1). IVC area, indexed IVC area, I:A ratio, pericardial thickness, RV area and indexed RV area were significantly different in patients who underwent pericardiectomy compared to those who did not (Table 1). Pericardiectomy was significantly associated with pericardial enhancement (p=0.011) as well as septal bounce (p<0.0001). The odds ratio (OR) for undergoing pericardiectomy in patients with septal bounce was 289 (95% confidence interval (CI) (16.681, 5007). Using ROC analysis, the area under the curve (AUC) and 95% CI for the prediction of pericardiectomy was 0.968 (0.92, 1.00) for IVC area, 0.932 (0.86, 1.00) for indexed IVC area and 0.963 (0.91, 1.00) for I:A ratio (Figure 1). An IVC area of 7.0 cm^2^ had 92% accuracy (sensitivity=94%, specificity=89%), an indexed IVC area of 3.4 cm^2^/m^2^ had 86% accuracy (sensitivity=94%, specificity=78%) and a I:A ratio of 1.8 had 92% accuracy (sensitivity=89%, specificity=94%).

**Table 1:** Quantitative CMR measurements and p-values from the two-sample t-test comparing patients who underwent pericardial stripping to those who did not.

	**Pericardium stripped** (n = 18)				**Not stripped** (n = 18)				
	**Mean**	**SD**	**Min**	**Max**	**Mean**	**SD**	**Min**	**Max**	**p-value**

IVC area (cm^2^)	9.59	2.59	6.3	14.6	5.02	1.36	3.0	7.5	<0.001*
Indexed IVC area (cm^2^/m^2^)	4.93	1.47	3.1	7.9	2.77	0.74	1.4	4.0	<0.001*
I:A ratio	2.56	0.76	1.4	4.0	1.28	0.27	0.7	1.8	<0.001*
Maximum pericardial thickness (mm)	5.50	2.77	2	12	1.72	0.57	1	3	<0.001*
RA AP diameter (cm)	5.17	1.15	3.2	7.9	5.00	1.21	3.1	6.9	0.664
RA TV diameter (cm)	4.81	0.91	3.4	6.4	4.99	1.18	2.7	8.4	0.605
RA area (cm^2^)	20.49	8.52	7.3	40.7	22.24	8.62	9.0	43.1	0.546
Indexed RA area (cm^2^/m^2^)	10.31	3.83	4.8	19.0	12.02	4.22	6.0	22.8	0.209
RV area (cm^2^)	15.86	6.31	9.3	29.7	22.11	6.38	13.6	34.0	0.006*
Indexed RV area (cm^2^/m^2^)	7.95	2.77	4.8	14.4	12.13	3.35	7.3	18.5	<0.001*

SD, standard deviation; Min, minimum value; Max, maximum value; RA, right atrium; AP, anterior posterior; TV, transverse; RV, right ventricle. *Denotes statistically significant result (p<0.05)

## Conclusions

Multiple CMR features are potential predictors of need for surgical relief of pericardial constriction. Measurement of IVC cross-sectional area is both sensitive and specific for the diagnosis of constriction and we propose an optimal cut-off value of 7 cm^2^ for absolute area or 3.4 cm^2^/m^2^ when indexed to BSA.

## Funding

None.

